# Droplet Digital PCR-Based Detection and Quantification of GyrA Thr-86-Ile Mutation Based Fluoroquinolone-Resistant Campylobacter jejuni

**DOI:** 10.1128/spectrum.02769-21

**Published:** 2022-04-12

**Authors:** Yi Luo, Wenting Zhang, Yiluo Cheng, Qin Lu, Yunqing Guo, Guoyuan Wen, Huabin Shao, Zhenyu Cheng, Qingping Luo, Tengfei Zhang

**Affiliations:** a Key Laboratory of Prevention and Control Agents for Animal Bacteriosis (Ministry of Agriculture and Rural Affairs), Institute of Animal Husbandry and Veterinary, Hubei Academy of Agricultural Sciences, Wuhan, China; b Hubei Provincial Key Laboratory of Animal Pathogenic Microbiology, Institute of Animal Husbandry and Veterinary, Hubei Academy of Agricultural Sciences, Wuhan, China; c Department of Microbiology and Immunology, Dalhousie University, Halifax, Nova Scotia, Canada; Health Canada

**Keywords:** FQ-resistant *Campylobacter jejuni*, ddPCR, detection, *gyrA* mutant

## Abstract

Fluoroquinolone (FQ)-resistant Campylobacter jejuni is a serious problem worldwide that limits effective treatment of infections. The traditional detection method depends on bacterial isolation and MIC testing, or traditional PCR, which is time-consuming and hard to identify the FQ-resistant C. jejuni in a high abundance wild-type background. This study aimed to develop a rapid and accurate ddPCR assay to detect FQ-resistant C. jejuni mutants based on the crucial resistance mutation C257T (Thr-86-Ile) in *gyrA*. Our ddPCR *gyrA* assay showed high specificity and accuracy. Sanger sequencing and the qPCR assay could only recognize *gyrA* mutant sequences when the ratios of wild-type/mutant were 1:1 or 10:1, respectively. Our ddPCR *gyrA* assay was able to detect *gyrA* mutant sequences in the mixtures with up to at least 1000:1 wild-type/mutant ratios, which suggested a significant advantage to distinguish the low mutant signal from the wild-type background. We further monitored the occurrence of *gyrA* mutations under ciprofloxacin pressure using our ddPCR *gyrA* assay, and clearly showed that the transition of a dominant C. jejuni subpopulation from wild-type to *gyrA* C257T mutant, resulting in FQ-resistance. We tested 52 samples from live chickens and retail chicken meat and showed that four samples contained wild-type/mutant mixtures comprising 1.7%, 28.6%, 53.3%, and 87.0% *gyrA* C257T mutants, respectively. These results demonstrated that the ddPCR *gyrA* assay was a highly sensitive alternative method to distinguish and quantify FQ-resistant C. jejuni infections that could help guide the appropriate use of FQs in clinical practice.

**IMPORTANCE** Campylobacter jejuni is considered to be the leading cause of human bacterial gastroenteritis worldwide, and fluoroquinolones (FQs) are the main choices for the treatment of bacterial gastroenteritis in clinical practice. In theory, antimicrobial susceptibility testing should help us to choose the most appropriate drugs for the treatment. However, to test the susceptibility of C. jejuni to FQs, the standardized method is bacteria isolation and MIC measurement, which will take more than 4 days. In addition, a low abundance of FQ-resistant C. jejuni is also hardly distinguished from a high abundance of wild-type background in the mixed infection. Therefore, the development of rapid and accurate detection technology for FQ-resistant C. jejuni is very important. This study provided a ddPCR *gyrA* assay, which is a highly sensitive alternative method to distinguish and quantify FQ-resistant C. jejuni infections that may help guide the appropriate use of FQs both in veterinary and human clinical practice.

## INTRODUCTION

Campylobacter jejuni is one of the major foodborne pathogens responsible for human bacterial gastroenteritis (1, 2), accounting for approximately 500 million infections per year globally (3). C. jejuni widely colonizes the intestinal tract of wild and domesticated animals and birds, and contaminated meat products are the main source of human infection (4). The detection of this pathogen mainly depends on bacterial isolation and PCR testing (5, 6). In clinical practice, fluoroquinolones (FQs) are one of the main choices for the treatment of bacterial gastroenteritis. To screen microbes for sensitivity to antimicrobials, the standardized method is MIC measurement according to the Clinical and Laboratory Standards Institute (CLSI), which involves bacterial isolation and identification, followed by the MIC test. However, for C. jejuni, this process takes more than 4 days, which hinders the choice of appropriate drugs during this time.

Epidemiological research indicates the widespread dissemination of FQ-resistant C. jejuni in humans and animals. In our previous studies, we identified a Thr-86-Ile mutation in GyrA that conferred FQ resistance in all C. jejuni isolates from chicken farms and retail chicken meat in central China (7, 8). High FQ resistance rates have also been reported in C. jejuni isolates from pigs (9, 10). An increase in the FQ resistance rate among C. jejuni isolates from patients has also been reported (11). For example, 74.3% of C. jejuni clinical isolates recovered from patients with acute gastroenteritis were found to be resistant to ciprofloxacin in Turkey (12). This rate was 48% in Chile (13) and more than 20% in the USA (11). An investigation of Campylobacter in a pediatric department showed that 77.4% of C. jejuni isolates from children were resistant to ciprofloxacin (14). The World Health Organization has reported that the emergence of FQ-resistant Campylobacter species is becoming a public health issue around the world (15). In theory, antimicrobial susceptibility testing should help us to choose the most appropriate drugs for the treatment of hospitalized patients (16). However, bacterial isolation and purification are required before testing, which are time-consuming bottlenecks for rapid responses. In addition, antibiotic-susceptible and -resistant isolates can be simultaneously present in an individual, but the low proportion of resistant strains is not easily detected in samples during the isolation process. Because of the wait time with antimicrobial susceptibility testing, FQs, such as ciprofloxacin, have been used to treat bacterial diarrhea before any resistance data, affecting therapeutic outcomes and promoting drug-resistant mutations among bacteria. Therefore, a rapid and sensitive method to identify FQ-resistant C. jejuni from clinical samples is required to evaluate the respective proportions of susceptible and resistant populations and the potential risk of treatment failure.

FQs target the DNA gyrase and topoisomerase IV of bacteria, inhibiting DNA replication, and transcription, and ultimately leading to cell death (17). Bacterial resistance to FQs is mainly related to chromosomal mutations in the quinolone resistance-determining regions (QRDRs) of DNA gyrase (encoded by the *gyrA* and *gyrB* genes), and partly to topoisomerase IV (encoded by *parC* and *parE*), as well as plasmid-mediated quinolone resistance (PMQR) genes, such as *qnr* and *aac*(*6′*)*-Ib-cr* (18, 19). In addition, the overexpression of efflux pumps also plays a role in the resistance to FQs (20, 21). In C. jejuni, the main reported mechanisms of FQ-resistance include mutation of the *gyrA* gene and alterations to multidrug efflux pumps. Mutations in the promoter of the *cmeABC* operon were shown to lead to overexpression of the CmeABC efflux pump and partly contributed to low-level resistance to antimicrobial agents in C. jejuni (7). A Thr-86-Ala mutation in GyrA (A256G in *gyrA*) has also been reported to confer resistance to nalidixic acid but not to FQs (22). However, high-level resistance to FQs (ciprofloxacin MIC >16 g/mL) in C. jejuni has only been reported to be caused by a Thr-86-Ile single mutation in GyrA (C257T in *gyrA*) (17, 23).

Droplet digital PCR (ddPCR) is a new technology that enables the absolute quantification of nucleic acids with high analytical sensitivity, specificity, and accuracy (24). To increase the sensitivity of resistance detection, a ddPCR *gyrA* assay, based on the resistant mutations of *gyrA*, was developed for the detection and quantification of FQ-resistant C. jejuni in this study.

## RESULTS

### Sanger sequencing of the *gyrA* QRDR of the wild-type/mutant mixtures.

**FIG 1:**
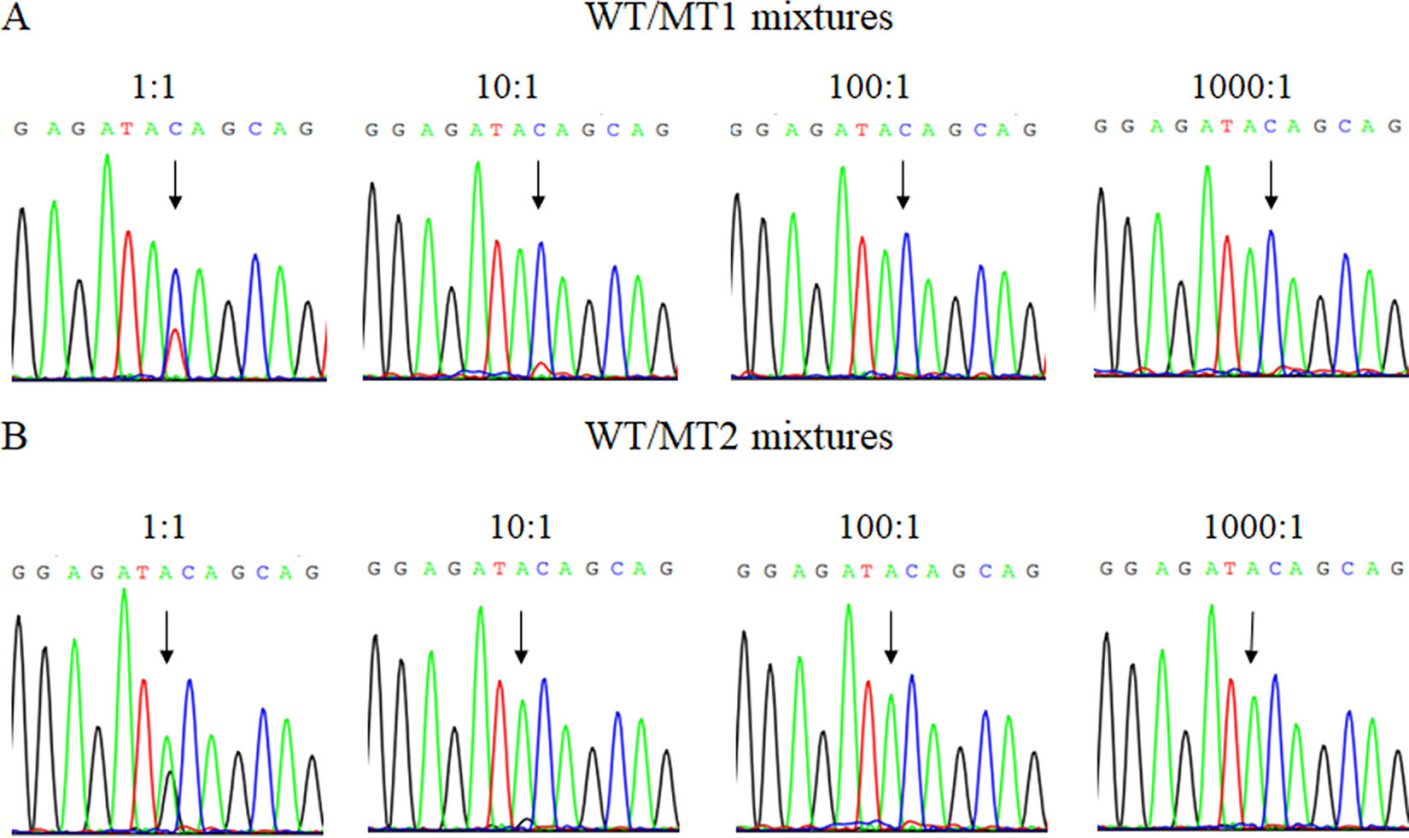
Sanger sequencing of the WT/MT mixtures. (A) WT and MT1 mixtures at 1:1, 10:1, 100:1, and 1000:1. (B) WT and MT2 mixtures at 1:1, 10:1, 100:1, and 1000:1. The arrows indicated the mutant peaks.

To assess the sensitivity of Sanger sequencing, the mixtures containing WT (pWT-*gyrA*) and MT1 (pMT1-*gyrA*) or MT2 (pMT2-*gyrA*) were analyzed. As shown in Fig. 1, C/T heterozygous peaks at the 257 site of *gyrA* in the mixture of WT and MT1, and A/G heterozygous peaks at the 256 site of *gyrA* in the mixture of WT and MT2 were detected at a 1:1 WT/MT ratio. In contrast, the MT1 and MT2 sequences were only occasionally detected at a 10:1 WT/MT ratio and were not detected at 100:1 and 1000:1 WT/MT ratios. This result suggested that the distinguishability of Sanger sequencing was at a 1:1 ratio of WT/MT.

### qPCR *gyrA* assay of the wild-type/mutant mixtures.

**FIG 2:**
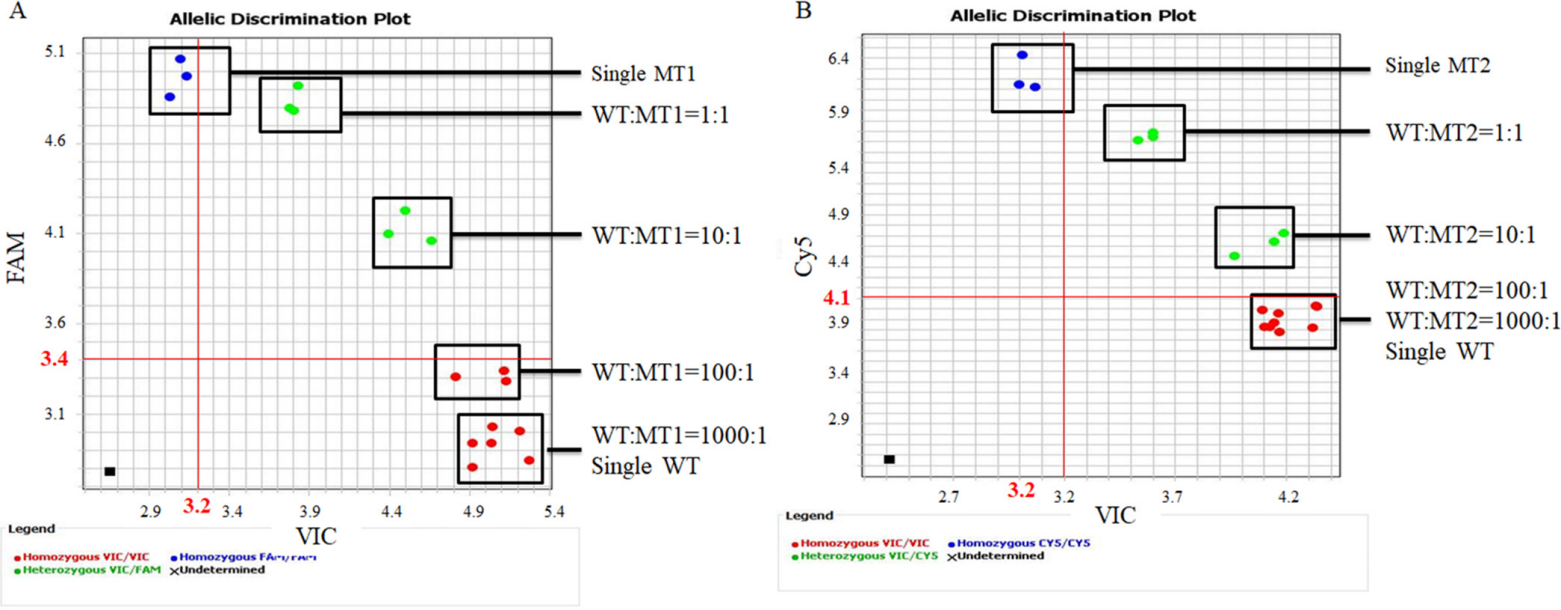
qPCR discrimination plots for the WT/MT mixtures. (A) WT and MT1 mixtures at 1:0, 0:1, 1:1, 10:1, 100:1, and 1000:1. (B) WT and MT2 mixtures at 1:0, 0:1, 1:1, 10:1, 100:1, and 1000:1. Black square indicates the negative control. The threshold is showed in red.

The specificity of the qPCR *gyrA* assay for detecting the WT (VIC), MT1 (FAM), and MT2 (Cy5) are shown in Table 1 and Fig. 2. The primers and probes are shown in Table 2. In Fig. 2B, a weak Cy5 fluorescence signal was detected when only WT was added to the reaction, which suggested a weak cross-reaction between the MT2 probe and the WT. To avoid the effects of weak fluorescence signals in the presence of mismatched targets, the thresholds of the fluorescence signals (shown in red in Fig. 2) were confirmed by detecting every single target. The qPCR *gyrA* assay was specific for detecting the WT, MT1, and MT2 sequences. No fluorescence signals were detected from species other than C. jejuni (Table 1).

**TABLE 1:** Strains used for specificity analysis of qPCR and ddPCR in this study

Strains^*a*^ or plasmids	qPCR			ddPCR		
	WT	MT1	MT2	WT	MT1	MT2
pWT-gyrA	+^*b*^	−	−	+	−	−
pMT1-gyrA	−	+	−	−	+	−
pMT2-gyrA	−	−	+	−	−	+
Campylobacter jejuni NCTC 11168 (WT)	+	−	−	+	−	−
Campylobacter jejuni WH31 (WT)	+	−	−	+	−	−
Campylobacter jejuni DY01 (MT1)	−	+	−	−	+	−
Campylobacter jejuni JSJ01 (MT1)	−	+	−	−	+	−
Campylobacter coli JSJ03	−	−	−	−	−	−
Campylobacter coli DY07	−	−	−	−	−	−
Escherichia coli DH5α	−	−	−	−	−	−
Escherichia coli CICC 21530	−	−	−	−	−	−
Salmonella pullorum CVCC 519	−	−	−	−	−	−
Salmonella enteritidis CVCC 540	−	−	−	−	−	−
Pasteurella multocida CVCC 1502	−	−	−	−	−	−
Staphylococcus aureus CVCC 545	−	−	−	−	−	−
Clostridium perfringens CVCC 79	−	−	−	−	−	−
Enterococcus faecalis CVCC 1927	−	−	−	−	−	−

a C. jejuni strains WH31, DY01, JSJ01, and C. coli strains JSJ03, DY06 were isolated in China and were kept in our lab. E. coli DH5α was kept in our lab. Other strains were purchased from the China Institute of Veterinary Drug Control.

b +, means test positive; −, means test negative.

To assess the sensitivity of the qPCR *gyrA* assay, the mixtures containing WT and MT1 or MT2 were analyzed. As shown in Fig. 2, both WT (VIC) and MT1 (FAM) or MT2 (Cy5) fluorescence signals were detected at 1:1 and 10:1 WT/MT ratios, respectively. In contrast, only the WT (VIC) fluorescence signal was detected at the 100:1 and 1000:1 WT/MT ratios. This result suggested that the distinguishability of the qPCR *gyrA* assay was at a 10:1 ratio of WT/MT.

### Validation of the ddPCR *gyrA* assay.

**FIG 3:**
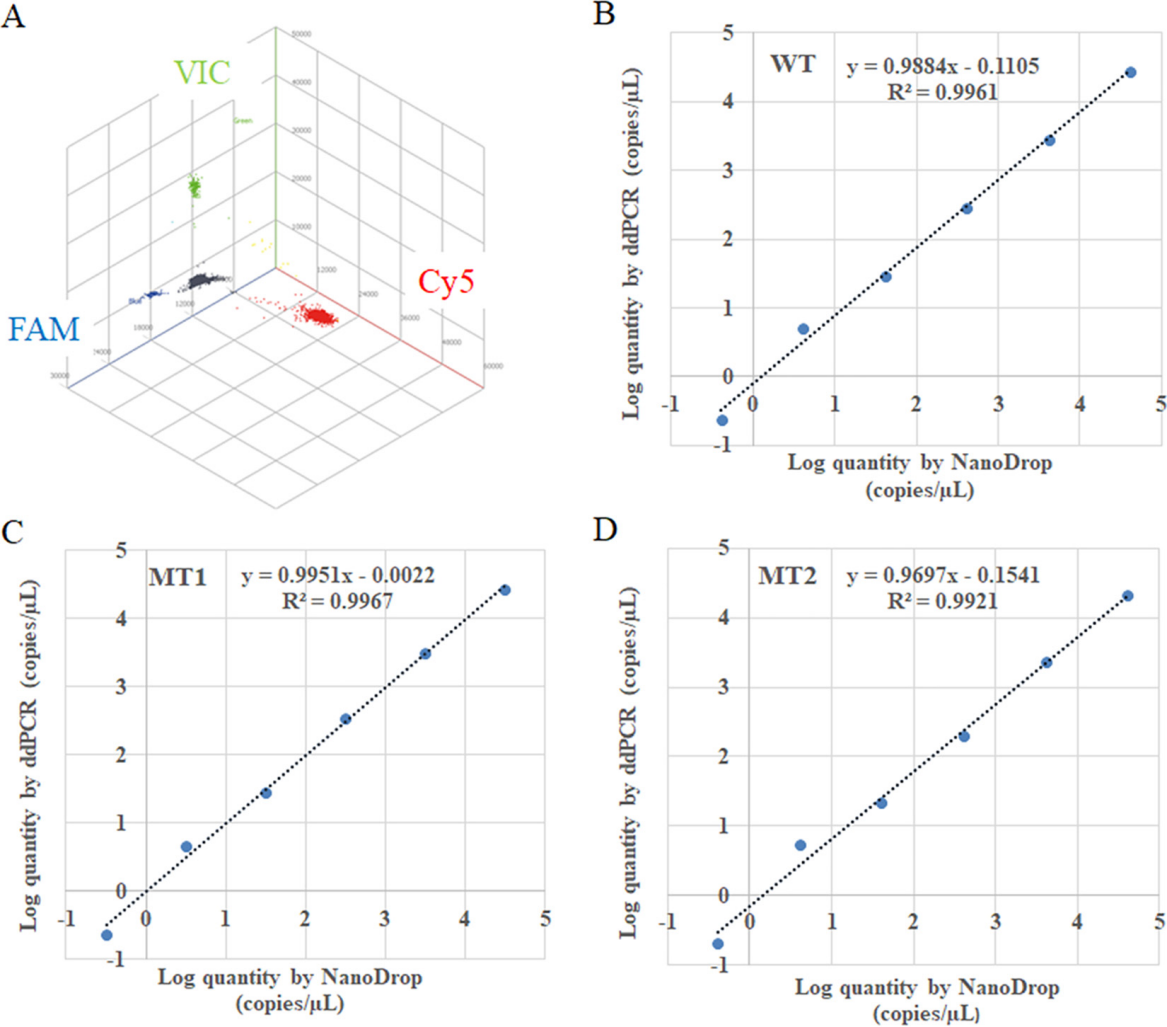
Validation of the ddPCR *gyrA* assay. (A) The three-dimensional diagrams for the ddPCR of the WT, MT1, and MT2 mixtures. Green dots represent wells with the VIC signal (corresponding to the WT); blue dots represent wells with the FAM signal (corresponding to MT1); red dots represent wells with the FAM signal (corresponding to MT2), and gray dots represent wells with no amplification signal. (B to D) The standard curves for the ddPCR are based on serial dilutions of the WT (B), MT1 (C), and MT2 (D).

The specificity of the ddPCR *gyrA* assay was evaluated by testing the constructed standard plasmids (Table 2), FQ-susceptible and FQ-resistant C. jejuni strains, and strains other than C. jejuni. As shown in Table 1, the ddPCR *gyrA* assay could detect and distinguish WT, MT1, and MT2 targets specifically, and the species other than C. jejuni were all negative. When testing the mixture of WT, MT1, and MT2 (1:1:1 ratio), the positive droplets from each plasmid template were in their corresponding fluorescent phase from the three-dimensional diagrams (Fig. 3A).

**TABLE 2:** Primers and probes used for qPCR and ddPCR assays in this study

Primer or probe names	Sequences and chemical modifications
C-gyrA-F	5′-CCCGTATAGTGGGTGCTGTT-3′
C-gyrA-R	5′-TCTTGAGCCATTCTAACCAAAG-3′
C-gyrA-WT-P	5′-VIC-ATGGAGATACAGCAGTTTAT-MGB-3′
C-gyrA-MT1-P	5′-FAM-ATGGAGATATAGCAGTTTAT-MGB-3′
C-gyrA-MT2-P	5′-Cy5-CCACATGGAGATGCAGCAGT-MGB-3′

Next, serial dilutions of WT, MT1, and MT2 were tested. The ddPCR *gyrA* assay showed good sensitivity and linearity at low copy number concentrations of targets (less than 3 × 10^5^ copies/reaction), and the concentrations provided by the software were close to the actual concentrations added to the reactions (Fig. 3B to D). For the WT, the slope was 0.9884, and the R^2^ was 0.9961. For MT1, the slope was 0.9951, and the R^2^ was 0.9967. For MT2, the slope was 0.9697, and the R^2^ was 0.9921. The minimum limit of detection reached the order of single-digit copies/reaction. However, when the concentrations of plasmids were at 10^6^ orders of magnitude of copies/reaction, the droplets clustered together and concentrations could not be obtained by ddPCR (unpublished data), which suggested that proper dilution was necessary.

### ddPCR *gyrA* assay of the wild-type/mutant mixtures.

**FIG 4:**
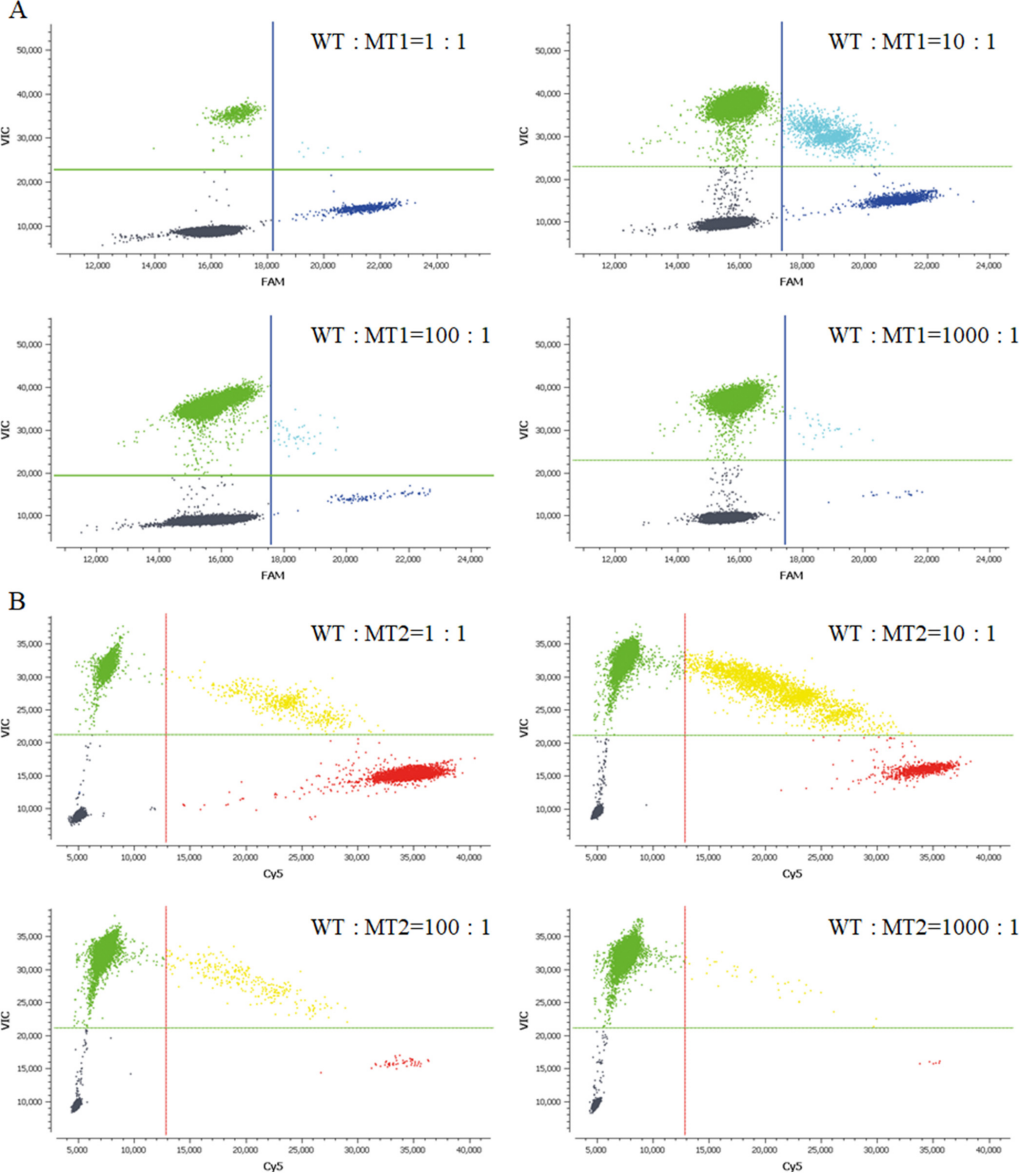
ddPCR *gyrA* scatterplots for the WT/MT mixtures: (A) WT and MT1 mixtures at 1:1, 10:1, 100:1, and 1000:1, respectively. (B) WT and MT2 mixtures at 1:1, 10:1, 100:1, and 1000:1, respectively. Gray dots represent wells with no amplification signal; green dots represent wells with the VIC signal (corresponding to the WT); dark blue dots represent wells with the FAM signal (corresponding to MT1); light blue dots represent wells with the FAM plus VIC signal; red dots represent wells with the Cy5 signal (corresponding to MT2), and yellow dots represent wells with the Cy5 plus VIC signal.

To assess the sensitivity of the ddPCR *gyrA* assay, the mixtures of WT and MT were analyzed. The percentages of MT in the mixtures were calculated using the formula: MT copies/(MT copies + WT copies). As shown in Table 3 and Fig. 4, when MT1 or MT2 was added to the WT at 1:1 to 1000:1 WT/MT ratios, MT1 or MT2 was detected in the mixture, and the percentages of MT were generally consistent with the original ratios of the mixtures. This result suggested that the distinguishability of the ddPCR *gyrA* assay reached a 1000:1 ratio of WT/MT at least, which means that 0.1% MT1 or MT2 sequences could be detected accurately in WT and MT mixtures.

**TABLE 3:** The ddPCR results for the *gyrA* wild-type and mutant mixtures

Mutants (mutation)	WT/MT ratio	No. of copies/μL						Mean % of mutants (SD)
		WT (VIC)			MT1 (FAM) or MT2 (Cy5)			
		Chip1	Chip2	Mean (SD)	Chip1	Chip2	Mean (SD)	
MT1 (C257T)	1:1	237.40	242.00	239.70 (3.25)	246.20	240.30	243.25 (4.17)	50.37 (0.77)
	10:1	2,062.00	2,215.00	2,138.50 (108.19)	201.70	184.00	192.85 (12.52)	8.29 (0.88)
	100:1	2,750.00	2,879.00	2,814.50 (91.22)	20.70	21.50	21.10 (0.57)	0.74 (0.004)
	1,000:1	2,429.00	2,460.00	2,444 (21.92)	5.22	7.37	6.30 (1.52)	0.26 (0.06)
								
MT2 (A256G)	1:1	272.30	283.90	278.10 (8.2)	239.10	250.40	244.75 (7.99)	46.81 (0.08)
	10:1	2,362.00	2,428.00	2,395.00 (46.67)	245.90	249.10	247.5 (2.26)	9.37 (0.09)
	100:1	2,784.00	2,840.00	2,812.00 (39.60)	20.90	20.20	20.55 (0.49)	0.73 (0.03)
	1,000:1	2,880.00	2,895.00	2,887.50 (10.61)	2.82	2.58	2.58 (0.17)	0.093 (0.01)

### Analysis of the *gyrA* mutations under ciprofloxacin pressure.

**FIG 5:**
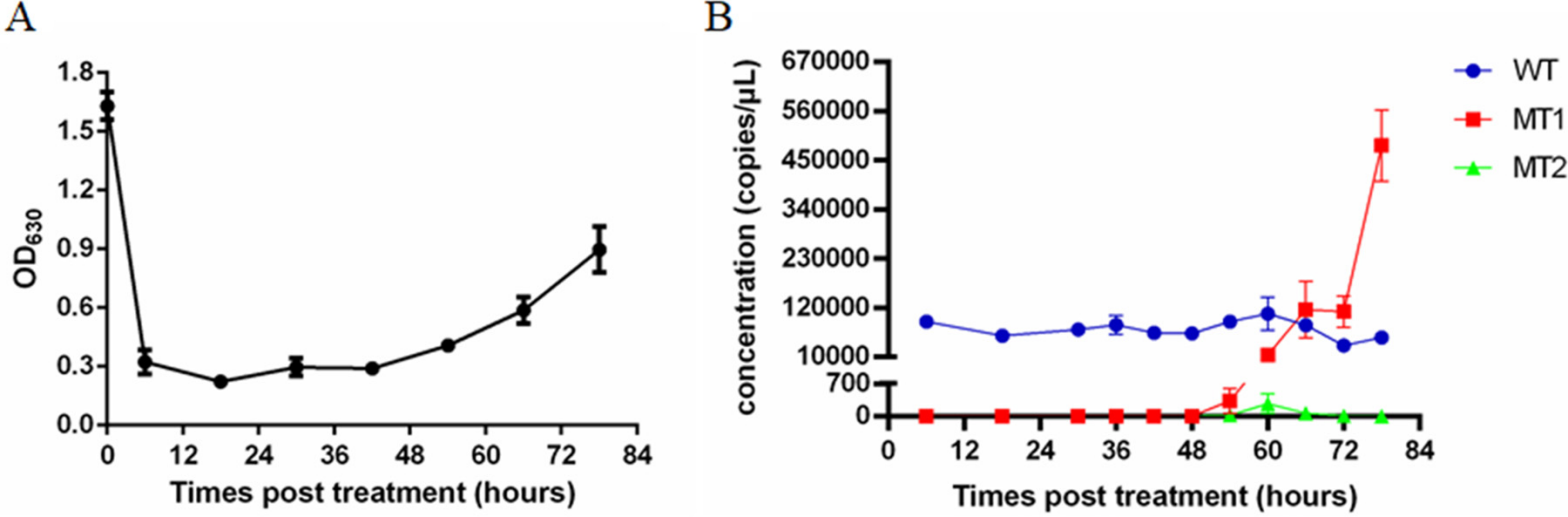
The transition of dominant C. jejuni subpopulations under ciprofloxacin pressure monitored by the ddPCR *gyrA* assay. (A) The survival curve of C. jejuni under ciprofloxacin pressure. (B) The transition of dominant C. jejuni subpopulations under ciprofloxacin pressure.

To further evaluate our ddPCR *gyrA* assay, the occurrence of *gyrA* mutations in FQ-susceptible C. jejuni strain NCTC 11168 (wild-type *gyrA*) under 10-fold MIC of ciprofloxacin (1 μg/mL) was monitored for 78 h. As shown in Fig. 5A, the optical density at 630 nm (OD_630_) reduced from 1.6 to 0.3 in the first 6 h after the addition of ciprofloxacin and then began to increase at 42 h posttreatment. As shown in Fig. 5B, the WT maintained a stable level ranging from 5.40 × 10^4^ to 1.07 × 10^5^ copies/μL during the beginning of the monitoring from the 6 h posttreatment, which suggested that the proliferation of wild-type C. jejuni was arrested. In contrast, 1.87 copies/μL of MT1 were detected 48 h after ciprofloxacin treatment, after which MT1 increased rapidly over the following 30 h and became the dominant *gyrA* type at 78 h (4.83 × 10^5^ copies/μL). During the monitoring, a few copies of MT2 irregularly appeared from 42 to 78 h. However, the abundance was lower than 2.71 × 10^2^ copies/μL, and there was no tendency to proliferate. Finally, the cultures were spread onto plates and 20 colonies were picked at 78 h posttreatment. Of these, 19 possessed the *gyrA* C257T mutation (MT1) and were ciprofloxacin-resistant, and only one was WT. This result further confirmed the identification results of the ddPCR *gyrA* assay, that MT1 was the dominant *gyrA* type in the cultures after 78 h of ciprofloxacin pressure.

### Analysis of anal swabs and retail chicken meat samples.

Fifty-two anal swab samples and retail chicken meat samples collected from chicken farms and markets were detected using the ddPCR *gyrA* assay. As shown in Table 4, one sample showed single VIC-positive droplets, indicative of the WT (FQ-susceptible C. jejuni strain). Twenty-two samples showed single FAM-positive (MT1) droplets, indicative of MT1 (FQ-resistant C. jejuni strain). No single Cy5 signals (MT2) were detected. Four samples showed both VIC and FAM-positive droplets, indicative of both WT and MT1 (mixture of FQ-susceptible and FQ-resistant C. jejuni strains), and the percentages of MT1 in these four mixture samples were 1.7%, 28.6%, 53.3%, and 87.0%, respectively. One sample showed both VIC and Cy5-positive droplets, indicative of both WT and MT2 (mixture of FQ-susceptible C. jejuni strains), and the percentage of MT2 was 66.4%. Further bacterial isolation and FQ susceptibility tests also confirmed our ddPCR *gyrA* assay results.

**TABLE 4:** The ddPCR results for detection of the clinical samples

ddPCR *gyrA* assays (no. = 52)	Samples (no.)	Resistant isolates		Susceptible isolates	
		No.	MIC (μg/mL)	No.	MIC (μg/mL)
VIC positive (WT)	1	0	—	1	0.125
FAM positive (MT1)	22	22	4-64^*a*^	0	—
Cy5 positive (MT2)	0	0	—	0	—
VIC&FAM positive (WT+MT1)	4	4	4-32	4	0.25-0.5
VIC&Cy5 positive (WT+MT2)	1	0	—	2	0.125/0.5^*b*^
Negative	24	0	—	0	—

a The MICs of these 22 isolates were in the ranges of 4 to 64 μg/mL.

b The MIC of WT isolate was 0.125 μg/mL, and the MIC of MT2 isolate was 0.5 μg/mL.

## DISCUSSION

C. jejuni is one of the most important foodborne pathogens, causing severe human gastroenteritis worldwide (3, 25). Antibiotic therapy is the main strategy for the treatment of C. jejuni infection, and the choice of drugs is particularly important for effective treatment. FQs (i.e., ciprofloxacin) are the most used antibiotics to treat acute bacterial diarrhea in humans and animals (17). Recently, FQ resistance in C. jejuni has increased because of the widespread use of this class of antibiotics (26, 27). In clinical practice, many cases are treated empirically with FQs without epidemiological or drug susceptibility analysis, which has limited the therapeutic effects of FQs. This is likely because such analyses are time-consuming using traditional methods (6). Therefore, in this study, we tried to establish a rapid and sensitive method for the identification of FQ-resistant C. jejuni from clinical samples.

The FQ resistance mechanisms of C. jejuni have been well studied (17). Unlike Escherichia coli and Salmonella, plasmid-mediated quinolone resistance genes have not been found in C. jejuni (18, 28). Although a variety of mutations in the DNA gyrase (encoded by *gyrA* and *gyrB*) and topoisomerase IV (encoded by *parC* and *parE*) have been identified in C. jejuni isolates by sequencing analysis, only a single point mutation, Thr-86-Ile in GyrA, is currently required for high-level resistance (ciprofloxacin MIC > 16 μg/mL) (29, 30). Asp90-Asn and Ala-70-Thr mutations in GyrA are rare and only confer intermediate-level resistance to FQs (29). Several studies have demonstrated that C. jejuni lacks the *parC* and *parE* genes, which strongly suggested that *parC* and *parE* cannot be a source of FQ resistance (31). In epidemiological investigations, the Thr-86-Ile mutation was detected in almost all high-level FQ-resistant C. jejuni isolates (7, 30). Based on this relatively specific characteristic of resistant mutations in the genome of C. jejuni, detection of FQ-resistant C. jejuni based on a molecular method was possible. Thus, we developed a ddPCR *gyrA* assay to detect and quantify FQ-resistant C. jejuni. We designed three probes, one targeting wild-type *gyrA*, one targeting the resistance mutation C257T in *gyrA* (Thr-86-Ile), and one targeting the *gyrA* A256G mutation (Thr-86-Ala), although this mutation was unusual and could not cause FQ resistance (22).

Sanger sequencing is the most used method to identify mutations in genes, and qPCR is another popular method. Therefore, we compared our ddPCR *gyrA* assay with Sanger sequencing and qPCR methods. With purified single samples, Sanger sequencing, the qPCR *gyrA* assay, and the ddPCR *gyrA* assay all showed good levels of detection. However, the ddPCR *gyrA* assay was able to accurately determine the copy number in each sample with good reproducibility (Fig. 3) even in the WT/MT mixtures (Table 3). Our ddPCR quantitative method did not require the determination of a standard curve, as for qPCR, or bacterial culture to provide viable counts. It is also worth noting that a weak cross fluorescence signal existed between the MT2 probe and WT *gyrA* target. Weak cross-reactivity is difficult to avoid in the detection of single nucleotide mutations based on qPCR using a TaqMan probe, so proof of threshold was necessary (32). However, cross-detection did not appear in the ddPCR *gyrA* assay. We inferred that because samples are divided into single droplets in the ddPCR system, any cross-fluorescence signals would be too weak in each droplet to be detected by the ddPCR system. Therefore, compared with the traditional detection method, the ddPCR *gyrA* assay is a fast and convenient bacterial quantitative method and can avoid nonspecific reactions.

When detecting mixed samples, *gyrA* mutant sequences were detected in mixtures of up to at least 1000:1 WT/MT ratios (0.1%) using the ddPCR *gyrA* assay. In contrast, Sanger sequencing and qPCR could only recognize *gyrA* mutant sequences at ratios of 1:1 or 10:1, respectively. Therefore, Sanger sequencing and qPCR might not be able to detect antibiotic-resistant subpopulations of C. jejuni in the early stage of infection. In principle, the ddPCR reaction mixture is divided into tens of thousands of independent units that are detected individually, so the sensitivity and discernibility of ddPCR are much higher than conventional PCR and qPCR methods. Therefore, our ddPCR *gyrA* assay offers a significant advantage for the detection of low-abundance FQ-resistant C. jejuni mutants, especially in mixed infections of FQ-resistant and susceptible strains. Recently, ddPCR was also used for detecting resistance mutations in Mycobacterium tuberculosis and Legionella pneumophila, reaching 1% to 0.1% distinguishability (32, 33). A maximum distinguishability of ddPCR of 0.001% could be reached using an improved method to obtain a higher number of individual droplets (24). Therefore, compared with the traditional method, the ddPCR *gyrA* assay has an obvious advantage in sensitivity and distinguishability.

The advantage of the high sensitivity of the ddPCR *gyrA* assay was also demonstrated by monitoring the occurrence of *gyrA* mutations in the wild-type C. jejuni strain under 1 μg/mL ciprofloxacin selective pressure. This concentration of ciprofloxacin has been reported to reflect drug metabolism after oral administration of ciprofloxacin in chickens (34). As shown in Fig. 5, the MT2 sequence only appeared occasionally, and the MT1-type mutant gradually became the dominant subpopulation during culturing in the presence of ciprofloxacin. This result further suggested that the ddPCR *gyrA* assay was useful for detecting antibiotic-resistant subpopulations of C. jejuni at an early stage of the resistance emergence. Based on the monitoring, we also determined the characteristics of FQ-resistant mutant outbreaks under FQ pressure. This regularity was consistent with the high prevalence rate of FQ-resistant C. jejuni harboring the *gyrA* C257T mutation in animals and humans (7, 10).

When the ddPCR *gyrA* assay was applied to clinical samples, it was clear that FQ-resistant C. jejuni harboring the *gyrA* C257T mutation was the main epidemic strain. Mixed infections with FQ-resistant and susceptible isolates were also frequently detected. It is worth noting that many clones were screened to detect the resistant isolates in the mixed samples, especially in the samples with a low proportion of MT1. This detailed work may not be feasible in the clinic, so resistant isolates may be easily neglected. Furthermore, the *gyrA* C257T mutation readily occurred after FQ treatment in C. jejuni. Therefore, our ddPCR *gyrA* assay may have important applications for detecting resistance mutations at an early stage, allowing for timely adjustment of the treatment regimen.

Although the ddPCR *gyrA* assay has remarkable advantages for the detection of FQ-resistant C. jejuni, it also has some limitations. One limitation is the maximum detection limit, which means that proper dilution may be required for detection in cases of severe infection. Another limitation is that it cannot distinguish dead bacteria from viable bacteria based on DNA detection. In addition, this assay is based on the GyrA Thr-86-Ile mutation. Although this is the main FQ resistance mechanism, which covered most of the FQ-resistant C. jejuni, a small number of resistant strains with other resistance mechanisms may be missed. Therefore, there is a need for continuous monitoring to detect the emergence of new FQ-resistant C. jejuni strains.

In conclusion, based on the reported crucial resistance mutation in *gyrA*, a ddPCR *gyrA* assay for detection and quantification of FQ-resistant C. jejuni was developed. Compared with the traditional method, the ddPCR *gyrA* assay could avoid nonspecific reactions well and had high distinguishability to recognize the FQ-resistant mutants. It was also convenient for bacterial quantification. Therefore, it has multiple advantages in the detection of FQ-resistant C. jejuni and may have important applications in clinical practice to help guide the appropriate use of FQs.

## MATERIALS AND METHODS

### Bacterial strains and culture conditions.

The strains used for specificity analysis in this study are listed in Table 1. C. jejuni NCTC 11168 was used as an FQ-susceptible control. The FQ-resistant strain C. jejuni DY01, containing a C257T mutation in *gyrA*, was used as an FQ-resistant control. C. jejuni DY01 was isolated from a chicken farm in Hubei, China (35) and kept in our laboratory. For specificity testing, 14 reference or clinical strains, including four C. jejuni strains and 10 strains of species other than C. jejuni were used. C. jejuni and Campylobacter coli strains were grown on Bolton broth (Oxoid, Basingstoke, UK) or modified charcoal cefoperazone desoxycholate agar (mCCDA) plates containing 1% Campylobacter growth and selective supplements (Oxoid) for 48 h at 42°C in air-tight jars containing AnaeroPack (Mitsubishi, Japan) to generate microaerobic conditions. Escherichia coli and Salmonella were grown in Luria-Bertani medium (LB, Oxoid) at 37°C. Pasteurella multocida and Staphylococcus aureus were grown in tryptic soy broth medium (TSB, BD, Sparks, MD, USA) at 37°C. Clostridium perfringens was cultured anaerobically in a Brain-Heart Infusion medium (BHI, BD) at 37°C. Enterococcus faecalis was cultured in a BHI medium at 37°C.

### Preparation of standard plasmids.

Genomic DNA of bacteria was extracted using the MiniBEST Universal Genomic DNA Extraction kit (TaKaRa, Dalian, China) according to the manufacturer’s instructions. An internal part (453 bp) of the QRDR region of *gyrA* was amplified from C. jejuni NCTC 11168 (wild-type *gyrA*, defined as WT) or C. jejuni DY01 (*gyrA* C257T mutant, defined as MT1) using forward primer (*gyrA*-F: 5′-GCCTGACGCAAGAGATGGTT-3′) and reverse primer (*gyrA*-R: 5′-TGAGGTGGGATGTTTGTCGC-3′), and then cloned into a pMD18-T vector (TaKaRa). Because no C. jejuni strain contained a naturally occurring A256G mutation in *gyrA* in our lab at the beginning of this study, the *gyrA* fragment containing the A256G mutation (defined as MT2) was synthesized and cloned into pMD18-T by Sangon Biotech (Shanghai, China). The three constructed plasmids were confirmed by DNA sequencing and were defined as pWT-*gyrA*, pMT1-*gyrA*, and pMT2-*gyrA*, respectively (Table 1). The concentrations of plasmids were measured using the NanoDrop spectrophotometer (Thermo Fisher Scientific, MA, USA).

### Isolation, identification, and FQ susceptibility testing of C. jejuni isolates.

Freshly collected swabs were stored in Cary–Blair modified transport media (Hopebio, Qingdao, China), and was then transported to our laboratory. The samples were resuspended in 0.5 mL PBS and inoculated into 5 mL Bolton broth containing Campylobacter growth and selective supplements, which was incubated for 24 h at 42°C under microaerobic conditions. Following this, 100 μL of the culture was spread onto mCCDA plates containing Campylobacter selective supplements and incubated for 48 h at 42°C under microaerobic conditions (36). Tentative C. jejuni colonies were further purified and identified by PCR targeting *hipO* as described previously (37). The MIC of ciprofloxacin of C. jejuni isolates was tested according to the CLSI guidelines (38). Strain C. jejuni ATCC 33560 was used for quality control of MIC tests.

### *gyrA* gene amplification and Sanger sequencing.

To assess the sensitivity of the PCR-sequencing approach, standard plasmid pWT-*gyrA* was mixed with pMT1-*gyrA* or pMT2-*gyrA* at 1:1, 10:1, 100:1, and 1000:1 ratios and were then tested by PCR and sequencing. The QRDR of *gyrA* was amplified by PCR using forward primer (*gyrA*-F: 5′-GCCTGACGCAAGAGATGGTT-3′′) and reverse primer (*gyrA*-R: 5′-TGAGGTGGGATGTTTGTCGC-3′′) as previously described (7). The 453-bp PCR products were sent to Sangon Biotech and sequenced using the same primers on a 3730xl DNA Analyzer system (ABI, USA). The sequences obtained were visualized and analyzed with DNAStar (Version 7.1, USA).

### Primers and probes designed for qPCR and ddPCR.

The primers and probes were designed based on the wild-type and mutant *gyrA* sequences of C. jejuni. The primer and probe sequences are listed in Table 2. The primers were used to amplify the core fragment of QRDR in *gyrA*, and the probes were designed to distinguish wild-type *gyrA* from two *gyrA* gene mutants of C. jejuni (MT1: C257T and MT2: A256G).

### qPCR validation and study of the wild-type/mutant mixtures.

The qPCR assays were performed using the LightCycler system (Roche, USA). Each of the qPCR mixtures (20 μL) contained 10 μL of Probe qPCR Mix with UNG (TaKaRa), 0.6 μM of each primer, 0.4 μM of each TaqMan probe, 2 μL of the tested DNA sample, and nuclease-free water. The qPCRs were performed with an initial denaturation step at 95°C for 30 s, followed by 40 cycles of denaturation at 95°C for 5 s, and annealing and extension at 60°C for 30 s.

Validation of the qPCR assays for the specific detection of *gyrA* WT, MT1, and MT2 sequences was performed by testing the pWT-*gyrA*, pMT1-*gyrA*, and pMT2-*gyrA* plasmids, and reference or clinical strains, including four C. jejuni strains and 10 strains of species other than C. jejuni (Table 1). To assess the sensitivity of the qPCR, 1 ng/μL of pWT-*gyrA* was mixed with 1 ng/μL of pMT1-*gyrA* or pMT2-*gyrA* at ratios of 1:1, 10:1, 100:1, and 1000:1. Therefore, the final concentration of the mixed plasmids (pWT-*gyrA* + pMT-*gyrA*) was 1 ng/μL. The mixtures were then tested by qPCR with three replicates.

### ddPCR validation and study of the wild-type/mutant mixtures.

The ddPCR *gyrA* assays were performed using the Naica System digital PCR platform (Stilla Technologies, Paris, France). Each of the ddPCR mixtures (25 μL) contained 12.5 μL of 2× PerfeCT qPCR ToughMix UNG (Stilla Technologies), 1.2 μM each primer, 250 nM each TaqMan probe, 100 nM fluorescein, and 2.5 μL of diluted DNA (i.e., 10 pg to 1 μg of DNA per reaction). The mixture was added to the sapphire chip, and the ddPCR assays were performed using the following program: 40°C for 40 min for droplet generation, followed by 95°C for 10 min, 40 cycles of 95°C for 15 s, 60°C for 30 s, and a final pressure release step for approximately 10 min. After PCR amplification, sapphire chips were scanned by the Naica Prism 3 Reader (Stilla Technologies). The resulting images were visualized and analyzed using the Crystal Miner software (Stilla Technologies).

The specificity of the ddPCR *gyrA* assay was tested using the standard plasmids, and reference or clinical strains (Table 1). To assess the linearity and detection limits of the ddPCR, 10-fold serial dilutions of each standard plasmid were tested with three replicates. To further assess the sensitivity of the ddPCR, pWT-*gyrA* was mixed with pMT1-*gyrA* or pMT2-*gyrA* at ratios of 1:1, 10:1, 100:1, and 1000:1, and these mixtures were tested by the ddPCR *gyrA* assay with three replicates.

### Mutations induced by the addition of ciprofloxacin and ddPCR detection.

To detect the occurrence of resistant mutations of FQ-susceptible C. jejuni under ciprofloxacin pressure, a susceptible C. jejuni strain NCTC 11168 was cultured to OD_630_ = 1.6 in Bolton broth under microaerobic conditions. The cultures were then centrifuged at 3000 g for 5 min to remove the medium. The pellets were resuspended in 200 mL fresh Bolton broth (OD_630_ = 1.6) with the addition of 1 μg/mL of ciprofloxacin and then cultured under microaerobic conditions. Every 6 h, OD_630_ was measured, 10 mL of culture was collected, and the DNA was extracted and 100-fold diluted. The mutations were detected and quantitated using ddPCR *gyrA* assays. To ensure that low copies of mutations were not missing, if the mutations were not detected, the undiluted DNA would be detected again. Three biological replications were performed. Finally, 10 μL of culture was collected at 78 h after ciprofloxacin pressure and diluted into 90 μL PBS. The diluent was spread on an mCCDA plate without ciprofloxacin and grown for 48 h at 42°C under microaerobic conditions. Following this, 20 colonies were picked randomly, and their *gyrA* were amplified by PCR and sequenced as described above.

### ddPCR *gyrA* assay on clinical samples.

Fifty-two samples were collected from chicken cloacas and the surface of retail chicken meat using swabs. The chicken cloacas swabs were collected from the 280 to 300 days old Chinese local chickens, which were ready to be sold and then butchered. The retail chicken meats were purchased from the markets. Freshly collected swabs were resuspended in 0.5 mL of PBS, followed by centrifugation at 12000 g for 2 min. The supernatant was removed, and the DNA of sediment was extracted using the DNeasy PowerSoil kit (Qiagen, Dusseldorf, Germany) according to the manufacturer’s instructions. For detection, 2.5 μL of DNA (approximately 10 ng/μL) was tested by a ddPCR *gyrA* assay, as described above. To further confirm the results of ddPCR *gyrA* assays, C. jejuni was isolated and purified from the collected samples, and ciprofloxacin susceptibility was tested as described above.
